# Gyroscope measurements of the precession and nutation of Earth’s axis

**DOI:** 10.1126/sciadv.adx6634

**Published:** 2025-09-03

**Authors:** K. Ulrich Schreiber, Urs Hugentobler, Jan Kodet, Simon Stellmer, Thomas Klügel, Jon-Paul R. Wells

**Affiliations:** ^1^ Research Unit Satellite Geodesy, Technical University of Munich, Munich 80333, Germany.; ^2^ School of Physical and Chemical Sciences, University of Canterbury, Christchurch 8140, New Zealand.; ^3^ Dodd-Walls Centre for Photonic and Quantum Technologies, Dunedin, New Zealand.; ^4^ Physikalisches Institut, Universität Bonn, Bonn 53115, Germany.; ^5^ Bundesamt für Kartographie und Geodäsie, Bad Kötzting 93444, Germany.

## Abstract

High-precision, Sagnac interferometry has long been proposed as a route to test fundamental questions in physics such as the magnitude of relativistic precessions (e.g., the Lense-Thirring effect). Although many elaborate experiments have been performed using, for example, matter wave interferometry or even quantum entanglement, none are within six orders of magnitude of the sensitivity and stability required to achieve such a measurement. We report on the operation of a free space ring laser gyroscope over a period of 250 days under an ambient pressure stabilizing vessel continuously in an unperturbed underground laboratory. Because we measure relative to local inertial space, the precession and nutation motion of Earth’s axis are intrinsically contained in the observations. It is demonstrated that optical interferometry, using an ultrastable cavity, yields an accuracy limit for rotation sensing of 48 parts per billion (i.e., picoradians per second), less than an order of magnitude away from the regime in which relativistic effects can be measured.

## Teaser

Inertial rotation sensing with a ring laser gyroscope provides the precession and nutation motion of Earth with high accuracy.

## INTRODUCTION

**Fig. 1.**
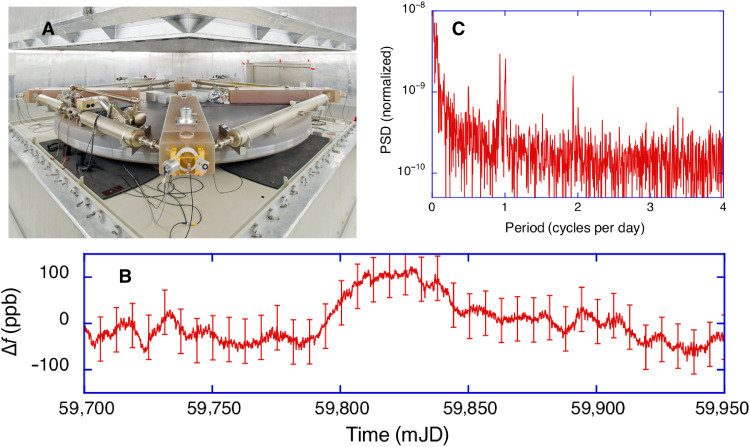
G ring laser in the underground laboratory and the current performance. The photo shows the body of the large gyroscope structure on a massive concrete monument, which is rigidly attached to bedrock. Above the instrument, one can see the top part of the ambient pressure stabilizing vessel (**A**). The diagram presents the remaining difference of the ring laser measurements against the IERS calculation when the complete transformation between ICRF and ITRF is performed. An SD of 48 ppb for the long-term variation is obtained (**B**). The spectrum of the measurement residuals only contains a few very small signal components at the level of 3 ppb in the diurnal and the semidiurnal band (**C**). mJD, modified Julian Date; PSD, power spectral density. (Photo credit: A. Heddergott/TUM)

The determination of the instantaneous rotation rate of Earth is one of the important tasks of space geodesy to monitor small perturbations of Earth’s rotation rate and the orientation of the spin axis of Earth in space. Such measurements reveal mass transport phenomena in the components of Earth system. Continuously updated Earth orientation parameters are necessary to keep the global navigation satellite systems (GNSS) accurate and the international terrestrial reference frame current. As such, it is a mission critical measurement for global navigation. Very-long-baseline interferometry (VLBI) and GNSS observations from a global network of stations are combined with Satellite Laser Ranging (SLR) and Doppler Orbitography and Radiopositioning integrated by Satellite (DORIS) to perform this task. All of these techniques are geometrical in nature and are based on light travel time measurements. By contrast, an optical Sagnac interferometer is an inertial measurement technique, exploiting the Sagnac effect, where two resonant narrow linewidth laser frequencies are excited in a ring cavity and propagate around the enclosed area in opposite directions. When the instrument is rigidly connected to Earth’s crust, the interferometer experiences a frequency shift (*1*–*3*), strictly proportional to the physical rotation rate. Making the enclosed area larger increases the sensitivity of the instrument for rotation. However, the mechanical stability of the sensor reduces at the same time, so that a careful balance of these effects has to be found. The G ring laser at the Geodetic Observatory Wettzell in Southern Bavaria consists of a square cavity with 4 m length on each side. It is monolithically constructed from the low expansion material Zerodur and located horizontally in an underground laboratory (*3*) as illustrated in Fig. 1A. Although classical Sagnac interferometers use an external light source and measure a phase difference of the two counterpropagating beams, the two laser beams in the active laser gyro experience a frequency difference of

$$\Delta f_{S}\left(t\right)=\frac{4A}{\lambda P}\Omega \left(t\right)\text{cos}\theta \left(t\right)$$ (1)

where A is the area circumscribed by the laser beams, P is the perimeter, λ is the optical wavelength in the cavity, and θ is the colatitude of operation. The unperturbed rotation rate Ω is Earth’s rotation vector with a rate of 2π/86,164.1 rad/s, corresponding to one revolution in one sidereal day. When the two laser beams are interfered with each other, the beat note $\Delta f_{S}$ is obtained with extremely high resolution, provided the cavity offers a narrow linewidth. For the G ring laser, we observe a cavity ring-down time of τ=1.2 ms, which translates into a quality factor of $Q=\omega \tau \approx 3.5\times 10^{12}$ , corresponding to a laser linewidth of about 7 μHz. The sensor sensitivity to rotations is smaller than 0.2 prad/s. Although sensitivity is straightforward to obtain by making the cavity large and using mirrors with extremely low loss, sensor stability, in particular for the long term, is much more difficult to achieve. This difficulty is not specific to laser gyros, and it applies, in general, to all ultrasensitive measurement devices. One prominent example is matter wave interferometry, which has been recently reported to reach an accuracy level of 10 parts per million (ppm) (*4*). Another approach uses quantum entanglement (*5*) and aims to measure general relativistic effects on entangled photons.

Applications in geophysics ultimately require a resolution and stability of 1 part in 10^9^ to accurately maintain the terrestrial reference frame with high stability because day-to-day perturbations in the rotation rate of Earth are very small but accumulate to notable seasonal changes over time. A closer inspection of Eq. 1 identifies two independent contributors to the total error margin. There is the scale factor 4A/(λP) stability and changes in the orientation θ of the gyroscope plane. The orientation itself has several contributors. For a single-component gyroscope, horizontally attached to the ground, the largest part is defined by the effective colatitude ( $\theta_{\text{lat}}$ ) of orientation. Variations in north-south tilt ( $\theta_{\text{tilt}}$ ), caused by solid Earth’s tides contribute to the measurement signal, as well as ground motion from earthquakes and microseismic activity. Diurnal polar motion ( $\theta_{\text{fn}}$ ) and the Chandler wobble ( $\theta_{\text{cw}}$ ) shift the instantaneous position of Earth’s rotation axis by as much as 8 m with respect to the body of Earth. This corresponds to a shift in colatitude and therefore shows up in the measurement signal. Inertial rotation sensing implies that the motion of Earth’s rotation axis in space, caused by precession ( $\theta_{\text{prec}}$ ) and nutation ( $\theta_{\text{nut}}$ ), produces a small measurement signal on an Earth fixed gyroscope. We therefore get

$$\theta \left(t\right)=\theta_{\text{lat}}\left(t\right)+\theta_{\text{tilt}}\left(t\right)+\theta_{\text{fn}}\left(t\right)+\theta_{\text{cw}}\left(t\right)+\theta_{\text{prec}}\left(t\right)+\theta_{\text{nut}}\left(t\right)$$ (2)

Intuitively, one would expect that the slow continuous precession and nutation motion of Earth’s axis is too small to matter for a gyroscope, rigidly strapped down to Earth, because the body of Earth moves along with the rotation axis without a relative change in orientation. However, because of the spin of Earth’s axis, the slow motion of the rotation axis of Earth in space produces a diurnal oscillation, which adds as an in-phase signal contribution to the diurnal polar motion signal. Taking this thought a step further toward the detection of relativistic quantities, namely, the de Sitter geodetic precession and the Lense–Thirring frame dragging, we would expect that these relativistic effects exhibit a similar behavior. The de Sitter term would be about a factor of 100 and the frame dragging term about 1000 times smaller than the precession of the Earth’s axis for a ground-based gyroscope (*6*, *7*). High-resolution, stable Earth rotation sensing is the obvious application for optical Sagnac interferometers. In this work, we provide evidence of the measurement of the precession and nutation of Earth and a thorough analysis of the current accuracy of the G ring laser.

## RESULTS

Over a period of 250 days, we have operated the gross-ring G in Wettzell in its underground vault (see Fig. 1A). We have applied all instrumental corrections to account for backscatter, nullshift, and drifts in the scale factor. We have corrected the measured rotation rate for colatitude and local sensor tilts to obtain globally valid values for Ω . At this stage, polar motion, variations of Ω , and the precession and nutation of Earth’s rotation axis itself are still contained in the data. A ring laser gyroscope is an inertial sensor, which means that every change in orientation of the normal vector on the laser plane with respect to the universe is reflected in the observations. Therefore, we have computed the full transformation from the International Celestial Reference Frame (ICRF) to the instantaneous ring laser position and orientation in the International Terrestrial Reference Frame (ITRF) in accordance with the conventions of the International Earth Rotation and Reference Systems Service (IERS) (*8*) and subtracted this from the preprocessed ring laser observations. This process is detailed in the Materials and Methods section. The remaining residuals are presented in Fig. 1B. The comparison of the full set of the ring laser measurements over the entire length of 250 days does not deviate from the combined results of the other space geodetic techniques by more than ±100 parts per billion (ppb). This includes the C04 time series and models for the forced nutation, also known as the diurnal polar motion or Oppolzer terms (*9*). It is important to note that the entire process does not contain any applied data filtering, curve fitting, or detrending. From the remaining residuals, we have calculated the single-sided power spectrum and the results are shown in Fig. 1C. The spectrum contains only tiny residual signals of up to 1 μHz in amplitude in the diurnal frequency band and 0.5 μHz in the semidiurnal band, corresponding to a level of 3 ppb in relative amplitude. These deviations can be caused either by a systematic error in the measurements, errors in the respective IERS models and the C04 time series, incomplete models, or a combination of all of them. Furthermore, it would potentially also contain the signal contributions from the relativistic effects. A rough estimate (*6*) would suggest an expected amplitude for the de Sitter term of 0.003 prad/s by the G ring laser, which is about one order of magnitude below the current noise floor. In terms of sensor resolution, there is about a factor of 2 between the gyroscopes in Gravity Probe B (1.35 × 10^−13^ rad/s) after 10 hours of integration and our ring laser (3 × 10^−13^ rad/s) over the same integration time. However, the bias stability for the mechanical gyros in orbit is much better than the optical ring cavity on ground.

**Fig. 2.**
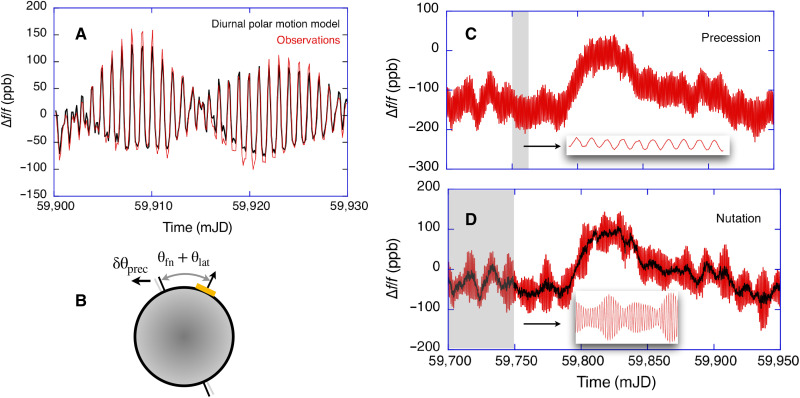
Effect of the precession and nutation of Earth on ring laser observations. The comparison of the ring laser observations to the diurnal polar motion model (*9*) exhibits a mismatch of 14% (**A**). The inertial sensing ring laser is sensitive to the motion of entire Earth in space including ${\delta \theta }_{\text{prec}}$ and not only the relative angular change $\theta_{\text{fn}}$ and $\theta_{\text{lat}}$ (**B**). When we do not account for the precession, we see the periodic diurnal effect on the ring laser measurements and a displacement of the residuals of −100 ppb. For better clarity, we show a 10-day blow up from the gray area in the inset (**C**). When only the nutation is excluded from the transformation, we obtain the corresponding signature from several frequency contributors (**D**) of the nutation signal. The inset of a 50-day section illustrates this. The exclusion of the nutation does not displace the ring laser residuals. We show this by adding the residuals of Fig. 1B in black to the display. The observed signals are all normalized to Earth’s rotation rate.

In our previous work, we have mainly concentrated on the improvement of the sensor precision (*3*, *10*). Here, we evaluate the accuracy that the device delivers and expand our analysis to include the precession of Earth, which is caused by gravitational forces acting on the slightly oblate body of Earth. It takes nearly 26,000 years for one cycle of precession to complete. Nutation has similar causes but acts on shorter periods with 18.6 years as the dominant frequency component. There are many more contributors, such as a prominent fortnightly period and more reaching even down to the semidiurnal regime. The effect of nutation in the diurnal band is a little smaller than that of the precession. When we compare the observed diurnal polar motion signal (see Fig. 2A) to the model calculations (*9*), we observe a notable underestimation of the actual measurements by about 14%. In our past analysis (*11*), we have not taken the precession and nutation motion of the rotation axis of Earth into account because it was thought to be too small. The precession causes a shift of the rotation axis of Earth by as little as 242 μrad (50 arc sec) per year, and the effect of nutation is an order of magnitude smaller. A stable high-resolution laser gyroscope is inertially referenced, so that it is not only the motion of the sensor relative to Earth’s rotation axis that matters but also the motion of the rotation axis of Earth with respect to the universe. This is sketched in Fig. 2B, where the motion of entire Earth in inertial space, denoted as ${\delta \theta }_{\text{prec}}$ is indicated in light gray. For the G ring laser, this corresponds to a signal at the level of 1 part in 10^7^ relative to the rotation rate of Earth. We present the diurnal signal contribution of the precession in Fig. 2C. It was generated by excluding only the precession from the ICRF to ITRF transformation. The precession also generates an offset of −100 ppb. Even the nutation effects make a notable contribution as we display in Fig. 2D, this time by excluding the nutation from the transformation. Unlike the precession, the nutation does not generate an offset. Instead, we see that the nutation signal is composed out of several frequencies. Although slightly counterintuitive, the slow precession and nutation signatures show up as signals in the diurnal band. It is the spin of Earth around its instantaneous axis of rotation that modulates these signals with a periodic diurnal signature, corresponding to a sidereal day. For better visibility, we also display a section 10 days long as an inset to Fig. 2C, where the precession-induced modulation is indicated for the gray shaded area. The inset in Fig. 2D has the appearance of an amplitude modulation and is 50 days long.

To compute the diagrams in Fig. 2 (C and D), we have used the Bernese GNSS Software (*12*) for the transformation of the sensor position in the ICRF to the location of observation in the ITRF (*13*). Apart from precession and nutation, the corrections contain forced nutation, the variable rotation rate of Earth, corrections for the offset of the mean solar time at the Greenwich meridian (UT1) and the universal time (UT), the polar motion, and the Chandler motion by including the C04 time series of the IERS. This study reports the quantitative observation of the precession and the nutation of Earth by a strapped down inertial rotation sensor.

A single-component ring laser gyroscope senses the combined effects of a large number of processes (*10*). Some are related to the motion of the sensor along with the body of Earth, as discussed above. We now focus on the hardware design and systematic biases related to the lasing process. The geometrical scale factor S=(4A)/(λP) requires corrections to account for the refractive index of the laser gas within the cavity, the Goos-Hänchen beam displacement due to penetration of the optical beam into the mirror coatings, dispersion, as well as wavefront distortion caused by the curvature of the mirrors (*14*). Together, these scale factor corrections are as large as 0.5 ppm. We use $S^{\prime }$ to denote that these corrections are applied. A bidirectional ring laser is a weakly coupled oscillator (*15*), subject to systematic frequency pulling and pushing, due to backscatter coupling ( $\Delta f_{\text{BS}}$ ) and the effect of a nullshift bias ( $\Delta f_{\text{NS}}$ ). We compute the correction to the Sagnac frequency $\Delta f_{S}$ as detailed in (*1*, *16*–*18*) and apply them to the observations

$$\Delta f_{S}\left(t\right)=\Delta f_{\text{obs}}\left(t\right)-\Delta f_{\text{BS}}\left(t\right)-\Delta f_{\text{NS}}\left(t\right)$$ (3)

Some of the measurement errors do not arise from the ring laser gyro itself but come from auxiliary sensors, such as the tiltmeters, used for the detection of slow (local) ground motion. Furthermore, variations in atmospheric pressure cause variations to the attraction bias of the tiltmeter pendulum, due to variable air mass density. This needs to be corrected as well. For the preprocessed dataset, we then obtain

$$\begin{array}{c} \Delta f_{S}\left(t\right)=S^{\prime }\left(t\right)n\cdot \Omega \text{cos}\left[\theta_{\text{lat}}\left(t\right)-\theta_{\text{tide}}\left(t\right)-\theta_{\text{tilt}}\left(t\right)-\theta_{\text{attr}}\left(t\right)\right]-\Delta f_{\text{BS}}\left(t\right)-\Delta f_{\text{NS}}\left(t\right) \end{array}$$ (4)

For a continuous measurement campaign of 250 days, preprocessed with Eq. 4 and the full transformation from the ICRF to the ITRF applied, we obtain measurement residuals for the rotation rate of Earth with an SD of 48 ppb, as shown in Fig. 1B. For this dataset, all measurements are within an interval of ±100 ppb.

**Fig. 3.**
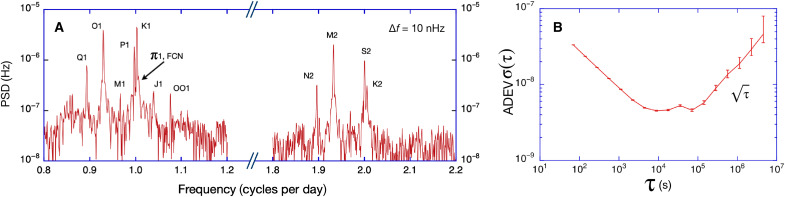
Ring laser observation of tidal signals. The spectrum of the raw ring laser observations around the diurnal and semidiurnal band exhibits a large number of tidal tilt signal contributions (**A**). The naming follows the standard convention (*19*). For the geodetic application, these signals are removed from the raw data by using a highly sensitive tiltmeter, which is collocated with the ring laser. The normalized ADEV ( $\delta \Omega /\Omega_{E}$ ) has been calculated by Stable32 over the entire set of observations (**B**). It levels out at 4 parts in 10^9^. For longer periods, this reduces as a random walk signal.

The G ring laser is only sensitive to rotations around the vertical. Equation 1 indicates that this leads to an ambiguity between the actual scale factor and the sensor’s latitude of operation. However, this ambiguity can be maintained experimentally, when both the scale factor and the orientation of the cavity are determined with high accuracy. We can rewrite Eq. 1 as

$$\Delta f_{S}\left(t\right)=\frac{4}{\lambda P}\cdot \Omega_{E}A\text{cos}\left[\theta \left(t\right)+\delta \right]$$ (5)

where all variables are defined as before and $\Omega_{E}$ is Earth’s rotation rate with a value of 2π/86,164.1 rad/s. The parameter δ accounts for small uncertainties in either the area *A* or the orientation θ . We obtain a value for δ through an initial measurement of the instantaneous rotation rate of Earth, and this fixes the initial orientation; for this measurement series, a value of $\delta =0.03{6^{\prime }}^{\prime }$ generates an offset of 150 ppb. By keeping track of the changes in orientation via a high-resolution tiltmeter and the perimeter of the gyroscope via the optical frequency in the rigid monolithic Zerodur cavity, a suitably stable condition is achieved. All other parameters in Eq. 5 are given with sufficient accuracy. Figure 3A illustrates the presence of harmonic constituents (*19*) of the diurnal and semidiurnal global tides in our long set of observations, which generate small local tilts. With a spectral resolution of 10 nHz in the diagram, a large number of different tidal constituents are resolved. These are identified by name in Fig. 3A, using the standard convention. Apart from the tides, there is also the evidence of the spectral component of free core nutation (FCN). We convert these observed geophysical tilts to a correction of the Sagnac frequency and subtract them from the measurement. This process also takes lunisolar attraction effects on the tiltmeter into account. Last, we remove variable attraction effects from changing atmospheric density according to (*20*). When all effects from ground motion have been adequately modeled, there are no evident spectral components of tidal signals left. In our case, residual tilts persist at a level of 1 ppb (see Fig. 1C).

**Fig. 4.**
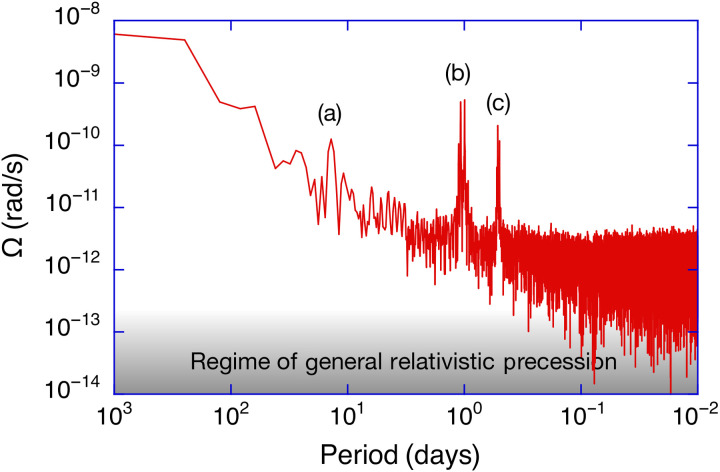
Broadband ring laser performance. The low-frequency performance of the G ring laser in continuous operation over a period of 250 days. There is no data fitting involved, and the relevant fortnightly (a), diurnal (b), and semidiurnal (c) geophysical signal bands are indicated. The measurements are shown as a function of signal frequency in the units of cycles per day. Ω is the observed sensitivity to rotation, and the regime of general relativistic signals is indicated.

A summary of all relevant error contributions are listed in Table 1. Backscatter coupling is the dominant error process, and it is time dependent. In our dataset, it causes a frequency pulling effect of up to 14.4 ppm of the observed Earth’s rotation signal. This error is corrected with good fidelity by a correction model (*16*) to remaining uncertainties below 50 ppb. The nullshift error, responsible for an asymmetry between the two counterpropagating laser beams is a magnitude smaller and reaches a value of −1.65 ppm. The remaining uncertainty after correction (*21*) is below 10 ppb. The scale factor of the G ring laser can be established with high accuracy (*14*). There are several contributors, and their combined uncertainty is below 20 ppb. The next two error sources to consider are the offset bias of the established tilt and the data error published by the IERS. Both error sources affect the accuracy estimate of the gyro but are not related to the ring laser gyro itself. It is important to note that we only use relative tilts. The tiltmeter, in the context of our observation, provides very small errors in the short term but develops a drift of 3 ppb over the length of our measurement series, which has been accounted for. Although we apply temperature corrections, we believe that some of the remaining signature of the observations, shown in Fig. 1B, is caused by the tiltmeter. For the diurnal polar motion, the Chandler wobble and the length of day signal, we apply the specified errors from the C04 series for Earth’s orientation data. The reported respective error levels of these signals are in the parts per billion regime and below the current resolution limit of the gyroscope. In Fig. 3C, we present the Allan Deviation (ADEV) of the entire 250 days dataset, calculated with Stable32 (*22*). In the first 3 hours, the observations average down as white frequency noise until a lower limit of $\Delta f_{S}/f_{S}=4\times 10^{-9}$ is reached, where the signal integration hits a flicker floor. The cause for that appears to be either Brownian motion (thermal) noise of the mirror coatings (*23*), residual gas scatter, or both. For integration times in excess of 2 days, the sensor drift takes over and the ADEV degrades slowly as random walk with a slope corresponding to $\sqrt{\tau }$. Figure 4 illustrates the current performance of our large ring laser interferometer over a wide range of frequencies. The ring laser gyro data still contain the observed geophysical signals in the fortnightly (a), the diurnal (b), and semidiurnal (c) geophysical band for illustration purposes. The regime where signals from general relativistic precessions are expected is less than one order of magnitude away.

**Table 1.** Error budget for the ring laser measurements. This table lists the known error sources and their relative effect on the observed Sagnac frequency and an estimate of the remaining uncertainty of each contributor. Tilt effects are listed separately. The scale factor instability is inferred from the perimeter variations through continuous optical frequency measurements using an optical frequency comb. The error margin of the tiltmeter was obtained from a calibration process. The necessary corrections for the scale factor result from refractive index, wavefront curvature, and beam steering as detailed in (*14*). The latitude and the scale factor are interchangeably connected for small values and highly sensitive. In the absence of two other gyroscope components, we chose to fix the latitude. The root mean square over all remaining potential uncertainties is estimated as 54 ppb, a value below our claim of achieved accuracy.

Source	Amount relative to the Sagnac frequency	Bias value	Uncertainty (ppb)	Reference
Latitude offset	5 × 10^−9^		2	–
Backscatter	1.44 × 10^−5^		50	(*16*)
Nullshift	−1.65 × 10^−6^		10	(*21*)
Scale factor	5.378 × 10^−7^		2	(*14*)
Scale factor instability		4 × 10^−12^	–	–
Atmospheric attraction		±4 × 10^−9^ (rad)	1	(*20*)
Tiltmeter		3 × 10^−6^ (rad)	13	
Diurnal polar motion	1.8 × 10^−7^		3	(*9*)
Chandler wobble	1.14 × 10^−5^		6	(*29*)
Length of day	3.4 × 10^−8^		6	(*29*)
Precession	2.8 × 10^−7^		1	(*8*)
Nutation	3.3 × 10^−8^		<1	(*8*)
Total			54	

## DISCUSSION

Earth’s rotation constitutes the link between ICRF and ITRF, but as Earth is a highly dynamic body, it is essential to obtain continuous observation of processes that influence its rotational motion. The monolithic single-component ring laser G has demonstrated long-term continuous observations with an SD of 48 ppb over periods as long as 250 days; this improves by one order of magnitude for periods shorter than 2.5 days. In terms of accuracy, the measurements do not deviate by more than ±100 ppb from the other techniques of space geodesy (see Fig. 1B), which corresponds to an accuracy estimate of 1 part in 10^7^ for the G ring laser. The root mean square of the tabulated measurement error budget is calculated as 54 ppb, and the backscatter correction, the tilt measurements, and the nullshift correction are the dominant contributors in the error budget. Subtle optical cross-coupling effects and the tiltmeter stability are the most likely causes for that. The precession and nutation rate of Earth’s axis, as well as the diurnal polar motion and the geophysical effects captured in the C04 time series and the IERS models, are detected with high fidelity and excellent agreement. The remaining discrepancy is mostly in the diurnal band and at the level of $\Delta f/f\approx 3.5\times 10^{-9}$ , and it is not clear whether this is a deficit in the sensor performance or the models. It may stem from ocean loading, zonal tides, nutation offsets, or general model deficits; this is subject to further investigation. Because we have only a single-component gyroscope, we are critically dependent on the tilt corrections. Long-term tilt effects from variable groundwater levels, a long-term tilt sensor drift, and atmospheric loading effects may still make a substantive contribution to the residuals of our observation. To clarify this point, a moderate improvement in the tiltmeter stability is required. The ring laser reaches a flicker floor at the level of $\Delta \Omega /\Omega \approx 4\times 10^{-9}$ . This can be due to thermal mirror coating noise or scatter noise contributions from residual gas in the cavity as suggested by model calculations; however, conclusive evidence is not currently available. In the long term, our gyro diverges with a random walk process. We also note that the ring laser is far more accurate in the short term. As VLBI provides outstanding long-term stability, a merger of the two techniques seems to be the logical next step.

## MATERIALS AND METHODS

**Fig. 5.**
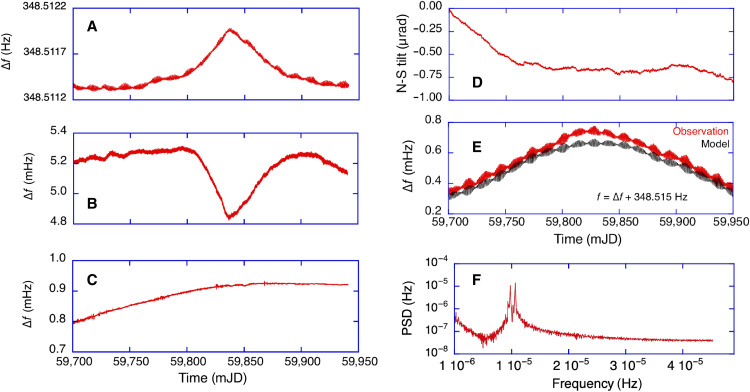
Illustration of the applied sensor corrections. The raw measurements from the data acquisition are shown (**A**). The backscatter corrections are computed and removed in the first step (**B**), followed by the nullshift offset correction (**C**). The effects from variations in the orientation of the laser plane, caused by local tilt, are taken from a tilt sensor and are removed in the next step (**D**). The transformation from the ring laser normal vector orientation, from the celestial reference frame ICRF, to the sensor position in the body fixed terrestrial system following the IERS conventions is taken with the mean value subtracted and is displayed and labeled “Model” in (**E**), whereas the corrected measurements minus the mean value and labeled as “Observations” are also shown in (E). Subtracting these curves from each other yields Fig. 1B. The spectrum calculated from the model (**F**) illustrates that most of the slowly changing signals in the ICRF end up in the diurnal band in the ITRF.

The ring laser gyroscope measures the beat note obtained by recombination of the two counterpropagating laser beams external to the cavity, as well as their respective intensities, continuously at a sampling rate of 2 kHz. The digitized measurements are binned into 1-min segments, and a frequency estimator based on the Hilbert transform is applied. The epoch of the measurement and the evaluated Sagnac frequency is then appended to the continuous observation file, together with the computed backscatter correction. Auxiliary data, in particular the temperature of the gyro body under the pressure stabilizing vessel, the atmospheric pressure inside and outside of the vessel, the plasma brightness, and the estimated tilts from several tiltmeters, complete each 1-min data record. During the analysis, the observations are averaged over 3 hours to reach the bottom knee of the ADEV (Fig. 3B) for the best resolution. All other auxiliary observations are averaged in the same way. The time series of the observed raw uncorrected Sagnac frequency marks the starting point of the analysis. The raw observations are presented in Fig. 5A.

In the first processing step, corrections for the backscatter coupling are applied in accordance with the procedure described in (*16*). The calculated corrections are shown in Fig. 5B. We can see that the backscatter coupling pulls the observed beat note down by about 5 mHz. As the last step of the laser-related corrections, we compute and apply the nullshift offset for the G ring laser, using a slightly modified version of the formalism detailed in (*17*, *18*, *21*, *24*) and the estimated correction series is displayed in Fig. 5C. The offset sensitivity in Eq. 5 for the value of δ is 150 ppb for a change of 0.036″ in latitude, and this had to be applied to obtain the result in Fig. 1C. Highly sensitive, inertial rotation sensors provide a local point measurement, whereas the measurement quantity of interest in geodesy is a global value. As such, local or regional ground motion can influence the measurements because Earth is deformable. In particular, local tilts at timescales between seconds and several months cannot be neglected. It is therefore very important to provide a rigid tie of the measurement sensor to the body of Earth. The G ring laser is large, grounded straight to bedrock with a massive concrete foundation and tilt corrected by a collocated tiltmeter (model: Lippman).

Local ground motion, caused by seasonal changes, variable groundwater levels, and atmospheric loading, is recorded by a tiltmeter on top of the monolithic gyroscope structure. The tiltmeter readout is corrected for attraction and ambient temperature effects. For improved mass attraction corrections, we use an additional model (*20*) that computes the effect of mass attraction by variable air density around the observatory. Figure 5D presents the sum of all these tilt corrections. It is important to note that these observations contain all the tilt effects caused by the solid Earth’s tides. Uplifts do not matter because the sensor is insensitive to translations. Once the tilt corrections are applied to the ring laser observations, almost all of the signals in the semidiurnal tidal band are removed from the ring laser measurements. Another tiny signal remains in the diurnal band of the ring laser observation. The largest amplitude of the discrete spectral components is 1 μHz or 3 ppb in the power spectrum of the ring laser beat frequency. This indicates a small mismatch between the observation and the applied models, but it is not clear whether it is an error from the gyroscope observation, the geophysical corrections, or a mismatch in both.

A ring laser gyroscope is an inertial sensor. It is sensitive to rotations around the normal vector on the ring laser plane and tilt effects of the normal vector only. As such, it is referenced to the local universe, i.e., it senses the full rotation vector of the sensor mounted on Earth’s surface with orientation given in the ITRF with respect to the ICRF. The total rotation vector sensed by the inertial sensor includes not only the rotation of Earth around its spin axis but also the rotation components from the precession and nutation of Earth’s axis in space as well as from the wobble of Earth’s body with respect to the spin axis. In our study, the rotation vector, represented in the ITRF, contains all rotation components according to the IERS Conventions (*8*). It specifically contains IAU 2006 precession and the nutation model according to IAU 2000A (*25*, *26*) together with nutation offsets measured by VLBI and published in the IERS EOP 20 C04 combined Earth’s rotation time series (https://datacenter.iers.org) that include FCN; they describe the motion of the Celestial Intermediate Pole (CIP) in the ICRF. The precession contributes notably to the measurement of Earth’s rotation sensed by the inertial sensor with a relative signal level of 10^−7^ in the diurnal frequency band. The nutation provides a smaller effect with a relative signal level of 10^−8^. In addition, the modeled total rotation vector includes geodetic precession to account for parallel transport of the rotation vector along Earth’s orbit in curved space-time caused by the Sun’s mass. The total rotation vector further includes the rotation of Earth around the CIP, including subdaily ocean tidal (*27*) and libration (*28*) effects in UT1. Last, it includes the rotation component of polar wobble, measured by space geodetic techniques and published in the IERS EOP 20 C04 series and including diurnal and semidiurnal forced terms accounting for ocean tides and forced nutation, describing the motion of the CIP in the ITRF. Actually, the forced nutation in the retrograde daily band (Oppolzer terms) provides the largest signal with a relative signal level a little below 10^−6^. It is shown in Fig. 5E together with the preprocessed ring laser observation. In the next step, we subtract the model from the observations. This provides the remaining residuals in Fig. 1B and we obtain an SD of 48 ppb. When the autopower spectrum is taken from the transformation model shown in Fig. 5E, we arrive at Fig. 5F, which also shows no signal of significance outside the diurnal band. From a physics point of view, there is no obvious limitation for the ring laser performance at this level of resolution. However, mirror coating noise (*23*) and residual noise from scatter within the gain medium inside the cavity are the most obvious candidates for the currently observed flicker floor in the G ring laser performance. A prominent peak is visible between day 59,800 and day 59,850 in Fig. 1B. It could be a small underestimation of the backscatter correction but may well have another physical cause. It is not related to the laser frequency in the cavity because most of that is a common mode effect. The optical frequency did not change by more than 15 MHz over the entire length of this dataset and furthermore does not show the same trend.

**Fig. 6.**
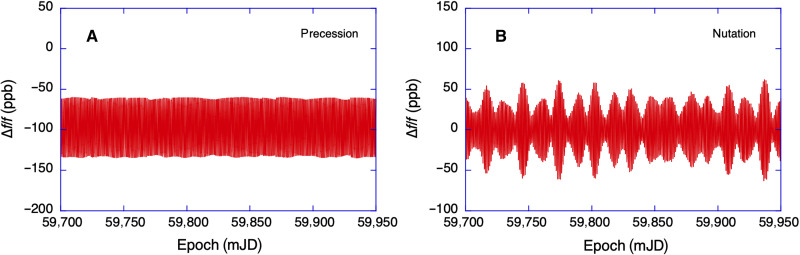
Extraction of the precession and nutation signals. Here, we present the remaining precession (**A**) and nutation (**B**) signal, contained in the ring laser observations. The signals were obtained from the observations, corrected for sensor effects and the subtraction of all geophysical signals with the exception of precession for the left diagram and nutation for the right diagram.

For the extraction of the precession and nutation signal components, we have taken the preprocessed observation time series (see Fig. 5E) and subtracted a model curve from the observations, which contained all the same signal contributors as before, with the exception of the precession. The result is shown in Fig. 2C. It is important to note that exclusion of the precession from the model causes an offset to the observation of about −150 ppb. This is to be expected because the spin axis of Earth is in progressive motion. We recall that the observations are all normalized to Earth’s rotation rate. When we also subtract the observation residuals from Fig. 1B, we obtain the unperturbed precession time series and this is presented in Fig. 6A. We have carried out the same procedure for the extraction of the nutation signal contribution and we obtain Fig. 6B. Here, we do not observe an offset. Instead, we see that the envelope of the remaining time series exhibits a beat pattern. It is composed of a large number of different frequencies, where the fortnightly period is the most obvious one for the length within our dataset.

## References

[1] F. Aronowitz, “The laser gyro” in *Laser Applications* (Academic Press, 1971), vol. 1, pp. 133–200.

[2] J. R. Wilkinson, Ring lasers. Prog. Quantum Electron. 11, 1–103 (1987).

[3] K. U. Schreiber, J.-P. R. Wells, Invited Review Article: Large ring lasers for rotation sensing. Rev. Sci. Instrum. 84, 041101 (2013). 23635174 10.1063/1.4798216

[4] R. Gautier, M. Guessoum, L. A. Sidorenkov, Q. Bouton, A. Landragin, R. Geiger, Accurate measurement of the Sagnac effect for matter waves. Sci. Adv. 8, eabn8009 (2022). 35687688 10.1126/sciadv.abn8009 PMC9187224

[5] R. Silvestri, H. Yu, T. Strömberg, C. Hilweg, R. W. Peterson, P. Walther, Experimental observation of Earth’s rotation with quantum entanglement. Sci. Adv. 10, eado0215 (2024). 38875336 10.1126/sciadv.ado0215 PMC11177943

[6] C. W. F. Everitt, D. B. DeBra, B. W. Parkinson, J. P. Turneaure, J. W. Conklin, M. I. Heifetz, G. M. Keiser, A. S. Silbergleit, T. Holmes, J. Kolodziejczak, M. Al-Meshari, J. C. Mester, B. Muhlfelder, V. G. Solomonik, K. Stahl, P. W. Worden, W. Bencze, S. Buchman, B. Clarke, A. Al-Jadaan, H. Al-Jibreen, J. Li, J. A. Lipa, J. M. Lockhart, B. Al-Suwaidan, M. Taber, S. Wang, Gravity Probe B: Final results of a space experiment to test general relativity. Phys. Rev. Lett. 106, 221101 (2011). 21702590 10.1103/PhysRevLett.106.221101

[7] I. Ciufolini, E. C. Pavlis, A confirmation of the general relativistic prediction of the Lense–Thirring effect. Nature 431, 958–960 (2004). 15496915 10.1038/nature03007

[8] G. Petit, B. Luzum, IERS Conventions (2010) (IERS Technical Note No. 36, IERS, 2010), pp. 1–179; https://www.iers.org/SharedDocs/Publikationen/EN/IERS/Publications/tn/TechnNote36/tn36.pdf?__blob=publicationFile\&amp;amp;v=1.

[9] A. Brzezinski, Contribution to the theory of polar motion for an elastic earth with liquid core. Manuscr. Geod. 11, 226–241 (1986).

[10] K. U. Schreiber, J. Kodet, U. Hugentobler, T. Klügel, J.-P. R. Wells, Variations in the Earth’s rotation rate measured with a ring laser interferometer. Nat. Photonics 17, 1054–1058 (2023).

[11] P. J. Mendes Cerveira, J. Boehm, H. Schuh, T. Klügel, A. Velikoseltsev, K. U. Schreiber, A. Brzezinski, Earth rotation observed by very long baseline interferometry and ring laser. Pure Appl. Geophys. 166, 1499–1517 (2009).

[12] R. Dach, S. Lutz, P. Walser, P. Fridez, *Bernese GNSS Software version 5.2 User manual* (Astronomical Institute, Univ. of Bern, Bern Open Publishing, 2015).

[13] K. U. Schreiber, U. Hugentobler, Ring laser transformation between ICRF and ITRF including applicable data files, Zenodo (2025); 10.5281/zenodo.15650985.

[14] R. B. Hurst, M. Mayerbacher, A. Gebauer, K. U. Schreiber, J.-P. R. Wells, High-accuracy absolute rotation rate measurements with a large ring laser gyro: Establishing the scale factor. Appl. Optics 56, 1124–1130 (2017). 10.1364/AO.56.001124 28158123

[15] G. E. Stedman, Ring-laser tests of fundamental physics and geophysics. Rep. Prog. Phys. 60, 615 (1997).

[16] R. B. Hurst, N. Rabeendran, K. U. Schreiber, J.-P. R. Wells, Correction of backscatter-induced systematic errors in ring laser gyroscopes. Appl. Optics 53, 7610–7618 (2014). 10.1364/AO.53.007610 25402929

[17] A. Beghi, J. Belfi, N. Beverini, B. Bouhadef, D. Cuccato, A. Di Vergilio, A. Ortolan, Compensation of the laser parameter fluctuations in large ring-laser gyros: A Kalman filter approach. Appl. Optics 51, 7518 (2012). 10.1364/AO.51.007518 23128698

[18] D. Cuccato, A. Beghi, J. Belfi, N. Beverini, A. Ortolan, A. Di Vergilio, Controlling the non-linear intracavity dynamics of large He–Ne laser gyroscopes. Metrologia 51, 97–107 (2014).

[19] National Oceanic and Atmospheric Administration, Tidal Harmonic Constituents, https://tidesandcurrents.noaa.gov/harcon.html?id=9414290.

[20] T. Klügel, H. Wziontek, Correcting gravimeters and tiltmeters for atmospheric mass attraction using operational weather models. J. Geodyn. 48, 204–210 (2009).

[21] A. D. V. Di Virgilio, N. Beverini, G. Carelli, D. Ciampini, F. Fuso, E. Maccioni, Analysis of ring laser gyroscopes including laser dynamics. Eur. Phys. J. D 79, 573 (2019).

[22] W. J. Riley, “A Test Suite for the Calculation of Time Domain Frequency Stability,” in *Proceedings of the 1995 IEEE International Frequency Control Symposium* (IEEE, 1995), pp. 360–366.

[23] K. U. Schreiber, J.-P. R. Wells, *Rotation Sensing with Large Ring Lasers: Applications in Geophysics and Geodesy* (Cambridge Univ. Press, 2023).

[24] A. D. V. Di Virgilio, N. Beverini, G. Carelli, D. Ciampini, F. Fuso, U. Giacomelli, E. Maccioni, A. Ortolan, Identification and correction of Sagnac frequency variations: An implementation for the GINGERINO data analysis. Eur. Phys. J. D 80, 163 (2020).

[25] N. Capitaine, P. T. Wallace, J. Chapront, Expressions for IAU2006 precession quantities. Astron. Astrophys. 412, 567–586 (2003).

[26] P. M. Mathews, T. A. Herring, B. A. Buffett, Modeling of nutation and precession: New nutation series for nonrigid Earth and insights into the Earth’s interior. J. Geol. Res. 107, ETG 3-1–ETG 3-26 (2002).

[27] R. D. Ray, D. J. Steinberg, B. F. Chao, D. E. Cartwright, Diurnal and semidiurnal variations in the Earth’s rotation rate induced by oceanic tides. Science 264, 830–832 (1994). 17794725 10.1126/science.264.5160.830

[28] A. Brzezinski, N. Capitaine, “Lunisolar perturbations in Earth rotation due to the triaxial figure of the Earth: Geophysical aspects,” in *Proceedings of the Journees 2001* (SYRTE–Observatoire de Paris, 2003), pp. 51–58.

[29] International Earth Rotation and Reference Systems Service, Paris Observatory, Earth Orientation Data, https://datacenter.iers.org/data/latestVersion/EOP_20_C04_0h_dPsi_dEps_1962-now.txt.

